# S2-3 Physical literacy in Portugal: assessment and intervention developments

**DOI:** 10.1093/eurpub/ckad133.011

**Published:** 2023-09-11

**Authors:** João Martins, Dora Carolo, João Mota, Marcos Onofre

**Affiliations:** Centro de Estudos em Educação, Faculdade de Motricidade Humana e UIDEF, Instituto de Educação, Universidade de Lisboa, Portugal; Centro de Estudos em Educação, Faculdade de Motricidade Humana e UIDEF, Instituto de Educação, Universidade de Lisboa, Portugal; School of Education, University College Cork, Ireland; Centro de Estudos em Educação, Faculdade de Motricidade Humana e UIDEF, Instituto de Educação, Universidade de Lisboa, Portugal

## Abstract

**Purpose:**

Physical literacy (PL) is being recognized as a key concept to set the foundation for health enhancing physically active lifestyles for all. Portugal is one of the European countries where PL is gaining momentum. This communication aims to present the Portuguese initiatives on PL assessment and intervention developments.

**Methods:**

In a first phase the Portuguese representatives on the EUROPLIT group identified diverse national sources of knowledge on PL research, practice and policy and produced a review. After a four-step data analysis conducted by two researchers on the reviews of 25 European countries, a quantitative survey integrating items that reflected the emerging themes from the overall content analysis was completed. The data related to the state of PL in Portugal is presented.

**Results:**

Portuguese PL research and pedagogical initiatives have been developed since 2016. FMH-ULisboa research group is leading the study and dissemination of PL through the development of PhD projects, the participation in European projects and the implementation of international scientific events. Regarding research efforts, it is highlighted the relevant advances on the study of PL assessment provided by the creation and validation of the Portuguese Physical Literacy Assessment instrument. Other Higher Education Institutions are also starting to create quality research on PL development. Intervention studies are missing, two recent innovative intervention projects were identified regarding PL development through blue spaces, and the effect of active breaks on PL and body composition. PL has recently been adopted in political documents and implemented in undergraduate, master, PhD level and Continuous Professional Development initiatives.

**Conclusions:**

The journey of implementing PL is beginning in Portugal. Conceptual, review studies and original research on PL assessment have been developed. Intervention studies are needed. Collaborative partnerships across education, sport and health sectors may be key to move PL forward.

